# 69350 Using Control Charts to Inform Public Health and Community Engagement during the COVID-19 Pandemic

**DOI:** 10.1017/cts.2021.715

**Published:** 2021-03-30

**Authors:** Moira Inkelas, Vladimir G. Manuel, Iheanacho O. Emerewa, Tony Kuo

**Affiliations:** 1 Fielding School of Public Health, University of California Los Angeles; 2 David Geffen School of Medicine, University of California Los Angeles; 3 Los Angeles County Department of Public Health and Fielding School of Public Health and David Geffen School of Medicine, University of California Los Angeles

## Abstract

ABSTRACT IMPACT: Demonstrate applicability of an underutilized method for showing variation that enables public health agencies to respond to the COVID-19 pandemic OBJECTIVES/GOALS: Enacting sensible public policies in the coronavirus disease 2019 (COVID-19) pandemic requires real-time data that civic and public health leaders can easily interpret and act on. This collaboration between a CTSA and a local health department sought a novel use of control charts to provide timely and interpretable data. METHODS/STUDY POPULATION: Healthcare and other industries use control charts to understand the behavior of processes and systems so they can intervene on them. The CTSA science team developed statistical process control charts at the neighborhood level to help illustrate their value for decision-making as the pandemic progresses. This method included accounting for congregate populations (skilled nursing facilities, correctional facilities) to produce data for the general public. RESULTS/ANTICIPATED RESULTS: Patterns in COVID-19 vary over time by neighborhood. Juxtaposing control charts with social characteristics of local areas in a dashboard format provides granularity for decision-makers and data for engaging communities in changing behavior. Annotating time series charts in real time connects events and local knowledge with observed data, which can help authorities and people to learn and act based on variations displayed by the control charts about disease outbreaks and cases. School districts are among those that could benefit from control charts with information about the school community and how COVID-19 spread is occurring. DISCUSSION/SIGNIFICANCE OF FINDINGS: Control charts have rarely been used in public health despite their ease of use and interpretability. This study demonstrates a novel approach to providing timely, accurate data that can support real-time decision-making of government and public health as well as school districts, businesses, and others.

